# Three-Dimensional Correlation Between Impacted Maxillary Canine Angulation and Root Resorption of Adjacent Lateral Incisors: A CBCT Study

**DOI:** 10.3390/diagnostics16132086

**Published:** 2026-07-03

**Authors:** Raed J. Abualfaraj, Hanadi Sabban

**Affiliations:** 1 Orthodontic Department, Faculty of Dentistry, King Abdulaziz University, Jeddah 21589, Saudi Arabia; rjabualfaraj@kau.edu.sa; 2 Oral Diagnostic Sciences Department, Oral Radiology Division, Faculty of Dentistry, King Abdulaziz University, P.O. Box 80209, Jeddah 21589, Saudi Arabia

**Keywords:** canine impaction, root resorption, cone-beam computed tomography, angulation, orthodontic diagnosis

## Abstract

**Background:** Impacted maxillary canines frequently cause external root resorption of adjacent lateral incisors, a complication that may compromise tooth survival and orthodontic outcomes. Cone-beam computed tomography (CBCT) enables precise three-dimensional assessment of canine position and resorption severity, yet the quantitative relationship between canine angulation and resorption remains incompletely characterized. **Objectives:** The objectives were to quantify the correlation between impacted maxillary canine angulation relative to the vertical reference line and the severity of adjacent lateral incisor root resorption using CBCT and to evaluate the influence of crown position on resorption prevalence. **Materials and Methods:** This retrospective cross-sectional study was conducted at the Department of Oral Radiology, King Abdulaziz University Dental Hospital (KAUDH), Jeddah, Saudi Arabia, using CBCT scans acquired between January 2018 and December 2023. CBCT scans of 262 patients with impacted maxillary canines were analyzed. A total of 108 impacted canines from 88 patients were evaluated. Canine angulation was measured as the acute angle between the canine long axis and a vertical reference line on coronal CBCT sections. Crown position was classified as palatal, buccal, or center. Lateral incisor root resorption was graded using the modified Kaley and Phillips classification (Grade 0–4), where Grade 4 represents side resorption. Statistical analyses included Spearman rank correlation, Mann–Whitney U test, chi-square test, and Kruskal–Wallis test with post hoc comparisons (α = 0.05). **Results:** Lateral incisor root resorption was detected in 17 of 108 canines (15.7%). Mean canine angulation was significantly higher in the resorption group (45.5° ± 21.4°) compared with the no-resorption group (32.7° ± 18.0°; Mann–Whitney U = 809.5, *p* = 0.019). Spearman correlation revealed a significant positive association between angulation and resorption grade (ρ = +0.243, *p* = 0.019). Canines angled ≥ 45° exhibited a 3.5-fold higher resorption rate (32.1% vs. 9.2%; OR = 4.66, Fisher’s Exact *p* = 0.012). Palatal crown position was associated with significantly higher resorption prevalence (25.5%) compared with center (5.1%) and buccal (11.1%) positions (χ^2^ = 7.258, *p* = 0.027). Unilateral impaction was associated with significantly higher resorption prevalence than bilateral (22.4% vs. 4.9%; *p* = 0.031). **Conclusions:** Larger canine angulation from the vertical reference line is significantly correlated with increased severity of adjacent lateral incisor root resorption. Palatal crown position, angulation ≥ 45°, and unilateral impaction pattern are important risk indicators. CBCT-based angulation measurement provides clinically relevant quantitative data to inform early intervention and treatment planning.

## 1. Introduction

Impacted maxillary canines represent one of the most common dental impaction anomalies, with a reported prevalence ranging from 1% to 3% in the general population [1,2]. The maxillary canine follows the longest and most tortuous eruption path of any permanent tooth, rendering it susceptible to developmental displacement and impaction [3]. When eruption is disrupted, significant complications may arise, including the displacement of adjacent teeth, the external root resorption of neighboring incisors, cystic transformation, and increased orthodontic treatment complexity [4,5,6]. Among these, external root resorption of the adjacent lateral incisor is particularly concerning, as it may progress silently until advanced stages when tooth prognosis is compromised [7,8]. Root resorption occurs when the impacted canine crown contacts the lateral incisor root during its aberrant eruption trajectory, initiating an inflammatory resorptive process that can lead to substantial root structure loss and, in severe cases, necessitate extraction with complex restorative rehabilitation [9,10,11]. Vertical growth patterns have also been associated with an increased prevalence of canine impaction, suggesting a multifactorial etiology [12].

Early detection and accurate three-dimensional localization of impacted canines are essential for implementing timely interceptive measures and minimizing the risk of irreversible root damage [13,14]. Conventional two-dimensional radiographic techniques, including panoramic and periapical radiographs, suffer from inherent limitations such as image magnification, distortion, and superimposition of anatomical structures, which reduce the diagnostic sensitivity for resorption and compromise measurement precision [15,16]. Cone-beam computed tomography (CBCT) provides high-resolution three-dimensional visualization with substantially lower radiation exposure than conventional CT and is recommended when three-dimensional assessment of impacted canine position or spatial relationships with adjacent structures are clinically justified [17,18]. Multiple studies have demonstrated CBCT’s superior sensitivity over panoramic radiography for detecting and grading lateral incisor root resorption [19,20] and its capacity for precise quantitative measurement of canine position and angulation in three dimensions [21,22].

The relationship between canine angulation and adjacent root resorption has been investigated in several studies, yet the findings remain inconsistent. Some CBCT-based investigations report significant associations between higher angulation and resorption severity [4,5,6], while others found no significant correlation using two-dimensional measurements [7]. The studies reporting no correlation were limited by the use of panoramic or periapical radiographs, which cannot reliably quantify three-dimensional angulation or detect early-stage resorption due to superimposition artifacts and reduced sensitivity [15,16]. Furthermore, previous CBCT studies lacked a standardized anatomically anchored angular reference, making inter-study comparisons unreliable. The present study addresses these shortcomings by employing a reproducible CBCT-based angulation measurement protocol referenced to the intermaxillary suture and anterior nasal spine (ANS) on coronal sections, enabling consistent quantification across cases. The influence of buccopalatal crown position on resorption risk also remains debated, with some studies reporting higher rates in palatal impactions [8,9] and others finding no significant difference by crown location [10]. A clinically applicable threshold angulation value beyond which resorption risk substantially increases has not been clearly established in the literature.

Objectives: The primary objective of this study was to quantify the correlation between impacted maxillary canine angulation measured on CBCT and adjacent lateral incisor root resorption severity. Secondary objectives were to (1) determine the resorption prevalence, (2) compare the angulation between canines with and without resorption, (3) evaluate the influence of the buccopalatal crown position on resorption prevalence, and (4) identify a threshold angulation value associated with increased resorption risk.

## 2. Materials and Methods

### 2.1. Study Design and Ethical Approval

This retrospective cross-sectional study was conducted at the Department of Oral Radiology, King Abdulaziz University Dental Hospital (KAUDH), Jeddah, Saudi Arabia. The study protocol was reviewed and approved by the Research Ethics Committee of the Faculty of Dentistry, King Abdulaziz University, Jeddah, Saudi Arabia (Approval No. 78-03-24, Date: 29 May 2024). The study was conducted in accordance with the Declaration of Helsinki. The requirement for informed consent was waived due to the retrospective nature of the study and the use of anonymized radiographic data.

### 2.2. Study Population and Sample Selection

**Figure 1:**
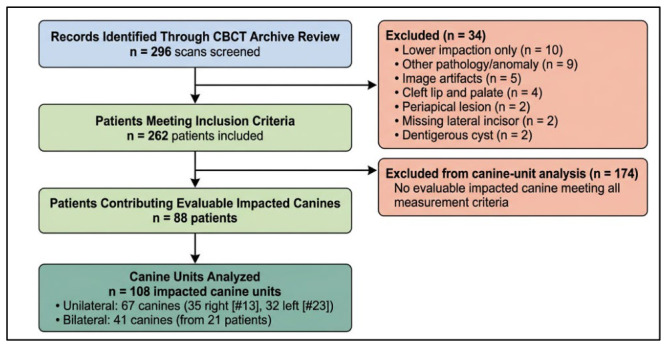
Flow diagram of study sample selection. Of 296 CBCT scans screened, 34 were excluded based on predefined criteria, yielding 262 eligible patients. A further 174 canine units were excluded for not meeting all measurement criteria, resulting in a final analytical sample of 88 patients contributing 108 impacted maxillary canine units.

CBCT scans acquired between January 2018 and December 2023 were systematically reviewed. The inclusion criteria were as follows: (1) presence of at least one impacted or partially impacted maxillary canine; (2) patient age ≥ 9 years; (3) diagnostic-quality CBCT with complete visualization of the maxillary anterior region; and (4) presence of the adjacent lateral incisor. The exclusion criteria included mandibular canine impaction only, congenital absence of the lateral incisor, dentigerous cyst or hyperplastic follicle (>3 mm), cleft lip and palate, periapical pathology affecting the lateral incisor, significant image artifacts, and prior orthodontic treatment involving the impacted canine. Of 296 CBCT scans initially identified, 34 were excluded (lower impaction only n = 10; other pathology/anomaly n = 9; artifacts n = 5; cleft palate n = 4; periapical lesion n = 2; missing lateral incisor n = 2; dentigerous cyst n = 2), yielding 262 included patients contributing 108 evaluable impacted canines from 88 patients. Of the 88 patients with evaluable impacted canines, 67 canines were from patients with unilateral impaction (35 right, 32 left), and 41 canines were from 21 patients with bilateral impaction. A patient-level flow diagram is provided in Figure 1 to illustrate the sample selection process.

### 2.3. CBCT Acquisition

All CBCT scans were acquired using standardized protocols with patients positioned according to the manufacturer guidelines. Datasets were reconstructed with isotropic voxel dimensions and reviewed using dedicated multiplanar reconstruction software. Imaging Equipment and Acquisition Parameters CBCT imaging was performed at the Oral Radiology Department, KAUDH, using a KaVo 3D eXam CBCT unit (KaVo Dental GmbH, Biberach, Germany). Scans were obtained with standardized parameters: 90 kV, 6.3 mA, exposure time 8.7 s, dose-area product 509 mGy/cm^2^.

Image Evaluation Datasets were reviewed using OnDemand3D software (Version 1.0.10.5385, Cybermed Inc., Seoul, Republic of Korea) in MPR mode in axial, sagittal, and coronal planes, following guidelines mandating systematic full-volume review.

### 2.4. Canine Angulation Measurement

**Figure 2:**
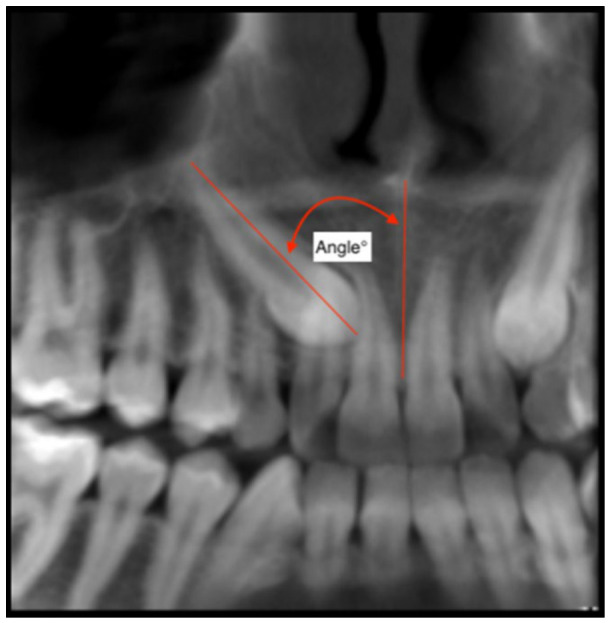
Representative coronal CBCT reconstruction demonstrating the standardized canine angulation measurement protocol. The vertical reference line is constructed along the intermaxillary suture passing through the anterior nasal spine (ANS). The long axis of the impacted canine is drawn from the cusp tip to the root apex; the measured angle (θ) is formed between this long axis and the vertical reference line.

Canine angulation was measured on coronal CBCT sections as the acute angle between the canine long axis and a vertical reference line constructed along the intermaxillary suture passing through the anterior nasal spine (ANS). The canine long axis was defined by connecting the midpoint of the incisal edge to the root apex. All angular measurements were performed by a single consultant orthodontist with more than 15 years of experience (RA) using digital measurement tools, recorded in degrees to one decimal place. A representative CBCT image illustrating the measurement protocol is provided in Figure 2.

### 2.5. Crown Position Classification

Buccopalatal crown position was classified on axial and coronal CBCT sections by a single oral and maxillofacial radiologist with more than 15 years of experience (HS) as follows: (1) palatal—canine crown positioned palatal to the alveolar ridge; (2) buccal—canine crown positioned buccal to the alveolar ridge; or (3) center—canine crown positioned within the alveolar ridge between buccal and palatal cortical plates.

### 2.6. Root Resorption Assessment

**Figure 3:**
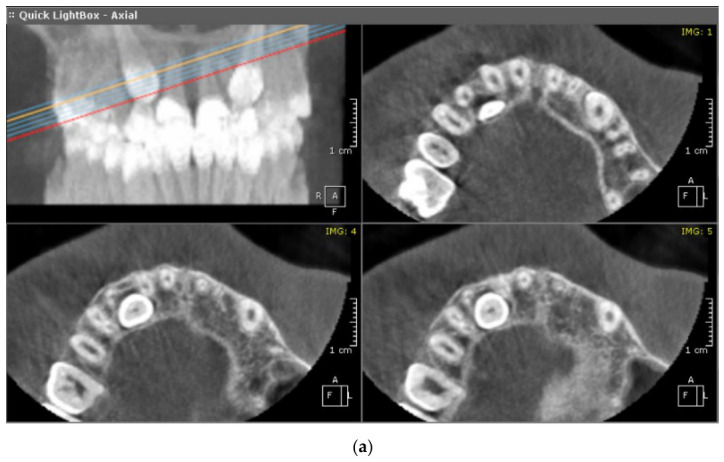

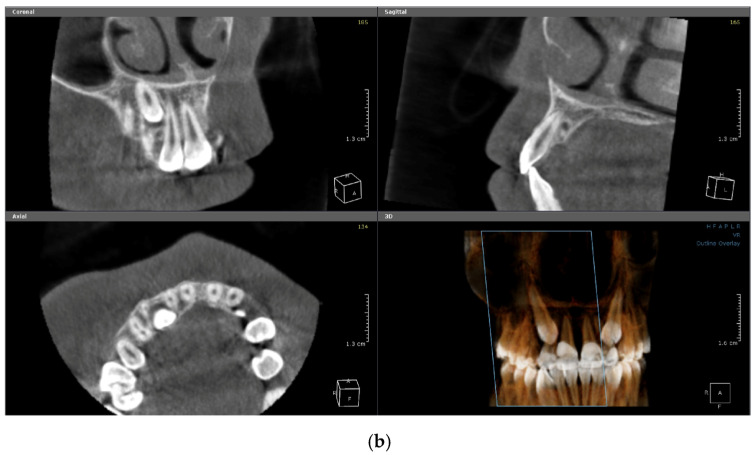
Cone beam CT (on-demand scan) of the same case. (**a**) Corrected axial sections (1 mm interval) showing the relationship between the lateral incisor and the impacted canine, with no root resorption. In the reformatted panoramic view, blue lines mark the stack of axial slices shown in the accompanying panels; red and yellow lines denote the superior and inferior boundary curves defining the sliced region. (**b**) MPR for same area with corrected coronal, and axial sagittal for the right canine also shows only side attachment with no resorption.

Lateral incisor root resorption was graded on multiplanar CBCT reconstructions by the same oral and maxillofacial radiologist (H.S., >15 years of experience) using the modified Kaley and Phillips classification [17]:

Grade 0: No resorption—smooth root contour (Figure 3).

**Figure 4:**
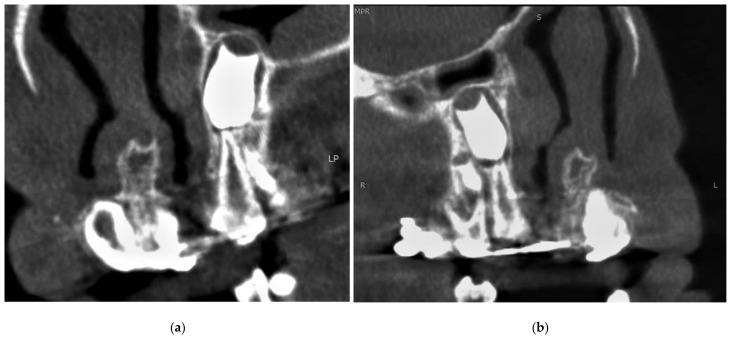
Corrected coronal CBCT sections illustrating lateral incisor root resorption adjacent to an impacted maxillary canine, graded using the modified Kaley and Phillips classification [17]. (**a**) Grade 1—slight apical blunting of the root apex. (**b**) Multiplanar reconstruction (MPR) of the same area—corrected coronal, sagittal, and axial views of the right canine—showing only side attachment with no root resorption. Orientation markers: LP, left posterior; S, superior; L, left; R, right.

Grade 1: Slight resorption—minor blunting of the root apex (Figure 4a).

Grade 2: Moderate resorption—apical resorption with up to one-quarter of root length lost (Figure 4b).

Grade 3: Severe resorption—apical resorption with greater than one-quarter of root length lost.

Grade 4: Lateral surface resorption—lateral or cervical surface resorption caused by direct contact of the impacted canine crown against the lateral surface of the lateral incisor root; a distinct morphological pattern separates from the apical resorption continuum of Grades 1–3.

### 2.7. Examiner Assignment

All canine angulation measurements were performed by a single consultant orthodontist with more than 15 years of clinical experience (R.A.) using the CBCT software’s (Version 1.0.10.5385) digital angle tool, with values recorded to one decimal place. Buccopalatal crown position classification and lateral incisor root resorption grading were independently performed by a single oral and maxillofacial radiologist with more than 15 years of experience (H.S.) on multiplanar CBCT reconstructions. Neither formal intra-examiner nor inter-examiner reliability testing was conducted for this study. This represents a methodological limitation; however, both examiners are senior consultants with extensive daily use of CBCT for orthodontic and dento-alveolar assessment, and clearly defined measurement protocols (Section 2.4, Section 2.5 and Section 2.6) were followed to minimize the subjectivity. Future prospective studies should incorporate intraclass correlation coefficients (ICC) for continuous measurements and Cohen’s kappa (κ) for categorical assessments to formally quantify the measurement reproducibility.

### 2.8. Missing Angulation Data

Valid angulation measurements were obtained for 93 of 108 impacted canines. The remaining 15 canines (13.9%) lacked a valid numeric angulation value. The following reasons caused missing measurement data: inadequate CBCT coverage precluding identification of both the cusp tip and root apex; raw entry recorded as “no CBCT”; presence of retained deciduous canine and lateral incisor complicating landmark identification; concurrent severe resorption of an adjacent central incisor altering local anatomy; a follow-up CBCT scan potentially acquired under different protocol conditions. These 15 cases were excluded from all angulation-dependent analyses (Spearman correlation, Mann–Whitney U test, 45° threshold analysis) but were retained in prevalence, crown position, and laterality analyses, where angulation was not a required variable.

### 2.9. Contralateral Side Assessment

The root resorption of lateral incisors on the contralateral (non-impacted) side was not evaluated in this study. For patients with unilateral canine impaction, only the lateral incisor adjacent to the impacted canine was assessed for resorption.

### 2.10. Statistical Analysis

Descriptive statistics were calculated for all variables. Normality was assessed using the Shapiro–Wilk test; angulation data were non-normally distributed (W = 0.9696, *p* = 0.029), necessitating non-parametric methods. The correlation between canine angulation and resorption grade was evaluated using Spearman rank correlation. Group differences in angulation were assessed with the Mann–Whitney U test. Associations between categorical variables were evaluated using chi-square tests or Fisher’s Exact Test as appropriate. Differences in angulation across crown positions were assessed using the Kruskal–Wallis test with post hoc pairwise Mann–Whitney comparisons. Statistical significance was set at α = 0.05.

## 3. Results

### 3.1. Sample Characteristics

Of 296 CBCT scans reviewed, 34 were excluded, yielding 262 included patients. Of these, 88 patients contributed 108 evaluable impacted canines. The mean patient age was 19.4 ± 9.6 years (range 9–57 years); 51 were female (58.0%) and 37 male (42.0%). Regarding laterality, 67 canines were from patients with unilateral impaction (35 right-sided [#13], 32 left-sided [#23]), and 41 canines were from 21 patients with bilateral impaction. The demographic and clinical characteristics are summarized in Table 1.

### 3.2. Canine Status and Crown Position

Among the 108 impacted canines analyzed, 63 (58.3%) were right maxillary canines (#13), and 45 (41.7%) were left (#23). The majority were fully impacted (n = 58, 53.7%), followed by partially impacted (n = 46, 42.6%), with four erupted but malpositioned canines (3.7%). Palatal positioning was most common (n = 51, 47.2%), followed by center (n = 39, 36.1%) and buccal (n = 18, 16.7%), consistent with the known predilection for palatal displacement in impacted maxillary canines.

### 3.3. Resorption Prevalence

Lateral incisor root resorption was detected in 17 of 108 canines (15.7%); here, 91 canines (84.3%) showed no resorption (Grade 0). The 17 resorption cases were classified as follows: Grade 1 (slight apical) n = 3 (2.8%), Grade 2 (moderate apical) n = 5 (4.6%), Grade 3 (severe apical) n = 3 (2.8%), and Grade 4 (lateral surface resorption) n = 6 (5.6%). Distinguishing the two morphological patterns, apical resorption (Grades 1–3) was present in 11 canines (10.2%) and lateral surface resorption (Grade 4) in 6 canines (5.6%). The full grade distribution is presented in Table 2. Representative CBCT examples of Grades 1 and 2 resorption are shown in Figure 3.

### 3.4. Angulation Measurements

**Figure 5:**
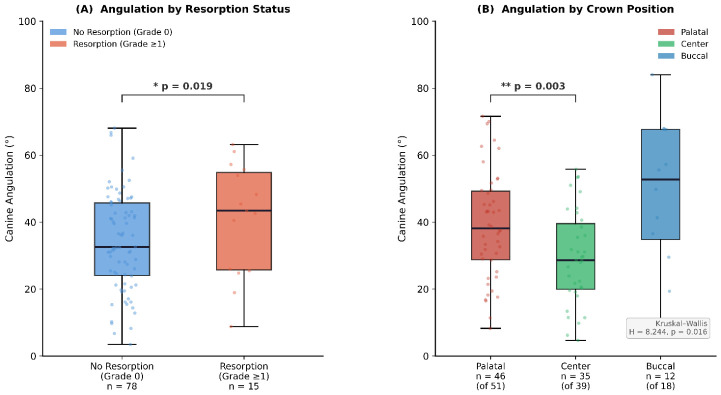
Box plots of canine angulation stratified by (**A**) resorption status and (**B**) crown position. Horizontal lines = medians; boxes = IQR (25th–75th percentiles); whiskers = min–max; circles = individual canines. (**A**) Resorption group (Grade ≥ 1, n = 15) vs. no resorption (Grade 0, n = 78); Mann–Whitney U = 809.5, * *p* = 0.019. (**B**) Palatal (n = 46), center (n = 35), buccal (n = 12); Kruskal–Wallis H = 8.244, *p* = 0.016; ** palatal vs. center post hoc *p* = 0.003. * *p* < 0.05; ** *p* < 0.01.

Valid angulation measurements were obtained for 93 of 108 canines. The angle was defined as the acute angle between the canine long axis and the vertical reference line, as described in Section 2.4. A larger angle therefore indicates a higher deviation of the canine long axis from the vertical reference line. The overall angulation ranged from 2.2° to 86.3° (mean 34.8° ± 19.1°, median 33.4°, IQR 19.1°–48.8°). Canines with resorption (n = 15 with valid angle) demonstrated significantly larger mean angulation (45.5° ± 21.4°, median 51.6°) than canines without resorption (n = 78; mean 32.7° ± 18.0°, median 30.0°; Mann–Whitney U = 809.5, *p* = 0.019). The mean angulation by resorption grade was Grade 0 (32.7° ± 18.0°), Grade 1 (53.0° ± 18.0°), Grade 2 (37.0° ± 32.6°), Grade 3 (52.5° ± 9.2°), and Grade 4 (43.6° ± 21.0°). The angulation also differed significantly by the crown position (Kruskal–Wallis H = 8.244, *p* = 0.016): palatal 39.1° ± 17.8°, buccal 38.8° ± 25.5°, center 27.7° ± 16.5°. Detailed angulation data stratified by resorption grade and crown position are presented in Table 3. Box plots and distribution data are shown in Figure 5.

### 3.5. Correlation Analysis

**Figure 6:**
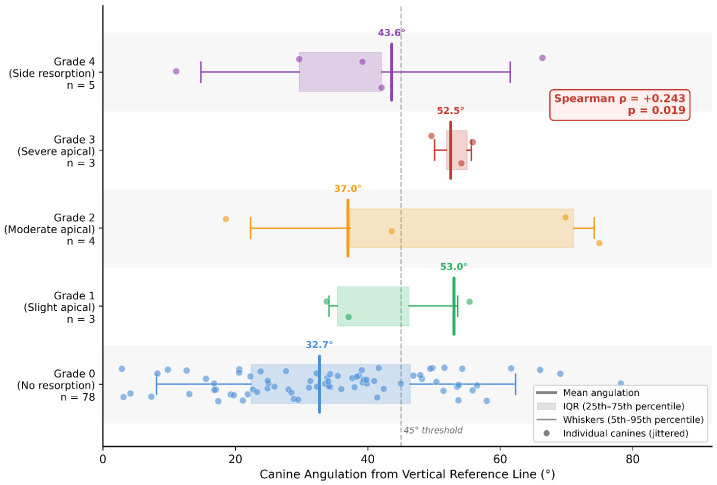
Distribution of canine angulation from the vertical reference line across resorption grades (Spearman’s ρ = +0.243, *p* = 0.019; n = 93). Each circle = one canine (jittered). Vertical bar = mean; box = IQR (25th–75th percentiles); whiskers = 5th–95th percentiles. The dashed vertical line marks the 45° threshold (resorption rate: 32.1% vs. 9.2% below; OR = 4.66, *p* = 0.012). Grade 0 = no resorption; Grade 1 = slight apical; Grade 2 = moderate apical; Grade 3 = severe apical; Grade 4 = side resorption.

Spearman rank correlation demonstrated a statistically significant, albeit modest, positive association between canine angulation and resorption grade (ρ = +0.243, *p* = 0.019), indicating a weak-to-moderate trend whereby canines with greater angulation from the vertical reference line tend to be associated with higher resorption grades. This correlation should be interpreted cautiously given the small number of resorption cases (n = 17) and the modest effect size. The threshold analysis revealed that canines angled ≥ 45° (n = 28) exhibited a resorption rate of 32.1% (9/28), compared with 9.2% (6/65) for those angled < 45°—a 3.5-fold increase in resorption risk above this threshold. Fisher’s exact test confirmed this difference was statistically significant (odds ratio = 4.66, *p* = 0.012), indicating that the odds of resorption were 4.66 times higher in the high-angulation group. The correlation between angulation and resorption grade is illustrated in Figure 6.

### 3.6. Crown Position and Root Resorption

The crown position was significantly associated with the resorption prevalence (χ^2^ = 7.258, df = 2, *p* = 0.027; Table 4). Palatal canines exhibited the highest resorption rate (13/51, 25.5%), followed by buccal (2/18, 11.1%) and center (2/39, 5.1%). Post hoc comparison confirmed significantly larger angulation in palatal versus center canines (*p* = 0.003), while the palatal vs. buccal (*p* = 0.737) and buccal vs. center (*p* = 0.283) differences were non-significant. Unilateral impaction was associated with significantly higher resorption prevalence (15/67, 22.4%) compared with bilateral impaction (2/41, 4.9%; χ^2^ = 4.634, df = 1, *p* = 0.031). The canine side (right 12.7% vs. left 20.0%; χ^2^ = 0.576, *p* = 0.448) and patient sex (χ^2^ = 1.023, df = 1, *p* = 0.312) were not significantly associated with resorption occurrence.

## 4. Discussion

This CBCT-based study demonstrates a significant positive correlation between impacted maxillary canine angulation and adjacent lateral incisor root resorption severity (Spearman ρ = +0.243, *p* = 0.019). Canines with resorption exhibited significantly larger angulation (mean 45.5° ± 21.4°) than those without (mean 32.7° ± 18.0°; Mann–Whitney U = 809.5, *p* = 0.019). The palatal crown position and angulation ≥ 45° emerged as the principal risk indicators. These findings provide quantitative evidence supporting the clinical utility of CBCT-based angulation measurement for risk stratification and treatment planning in patients with impacted maxillary canines.

The observed positive correlation aligns with biomechanical principles and the prior literature. Larger canine angulation increases the contact area and pressure between the canine crown and the lateral incisor root during eruption, promoting osteoclastic activity and progressive root resorption [5,9]. Simic et al. [5] reported that palatally displaced canines with larger angulation exhibited higher resorption rates, consistent with our results. Razeghinejad et al. [11] identified canine inclination as the strongest predictor of resorption severity in multivariate analysis and reported maximum resorption when the canine-to-lateral incisor angle was 30–60°; a range encompassing our 45° threshold. Similarly, Dagsuyu et al. [9] found that three-dimensional angular measurements on CBCT were significantly associated with the resorption grade, supporting the superiority of CBCT-based quantification over conventional radiographic approaches. Emad et al. [19] further corroborated these findings in a retrospective CBCT study demonstrating that higher canine angulation was associated with higher resorption severity, with a threshold effect observed around 45°. The mechanism involves sustained pressure activating osteoclasts and odontoclasts, resulting in progressive root loss [9,10]. Ucar et al. [10] reported that both the severity and frequency of root resorption increased proportionally with canine angulation, while Wang et al. [17] identified angulation as one of several independent risk factors in a multivariable model. In contrast, Kalavritinos et al. [7], using panoramic measurements, found no significant correlation, a discrepancy attributable to the inherent limitations of two-dimensional imaging in capturing the true three-dimensional angulation of the impacted canine.

The palatal crown position was associated with significantly higher resorption prevalence (25.5%) compared with center (5.1%) and buccal (11.1%) positions (*p* = 0.027), consistent with Al-Kyssi et al. [16], who reported a 3.2-fold higher resorption risk in palatally impacted canines. Yilmaz et al. [8] similarly reported that palatal canines had significantly higher resorption rates than buccally positioned canines. In our sample, palatal canines exhibited significantly greater angulation than center canines (39.1° vs. 27.7°; *p* = 0.003), suggesting that angulation mediates the relationship between palatal position and resorption risk. Lai et al. [6] and Oberoi and Knueppel [20] both demonstrated that the proximity and direct contact of the canine crown with the lateral incisor root were the strongest predictors of resorption—factors closely linked to both palatal position and higher angulation.

A novel finding of this study was the significantly higher resorption prevalence in unilateral compared with bilateral impaction (22.4% vs. 4.9%; χ^2^ = 4.634, *p* = 0.031; OR = 5.63). This may reflect that bilateral impaction cases are more frequently identified and monitored early due to the symmetrical presentation drawing clinical attention, whereas unilateral impactions may go undetected for longer, allowing more time for root resorption to progress. This finding has not been widely reported in the literature and warrants further investigation in larger prospective cohorts. The canine side (right vs. left) showed no significant effect on resorption prevalence (*p* = 0.448), consistent with the absence of inherent anatomical asymmetry in resorption risk, as reported by Guarnieri et al. [4] and Almuhtaseb et al. [22].

The overall resorption prevalence of 15.7% falls within the lower range of the reported rates (3–66%) [6,21]. This variability across studies reflects differences in diagnostic modalities—CBCT-based studies consistently report higher detection rates than conventional radiography due to superior three-dimensional visualization—as well as differences in population characteristics, age distributions, and resorption classification systems. Mitsea et al. [3] in a systematic review and meta-analysis reported a pooled resorption prevalence of approximately 38% using CBCT, highlighting that detection rates are substantially influenced by imaging modality.

A clinically important finding is the distinction between apical resorption (Grades 1–3, n = 11, 10.2%) and lateral surface resorption (Grade 4, n = 6, 5.6%). Lateral surface resorption is clinically significant because it may compromise root integrity at the cervical level, affecting periodontal attachment and long-term tooth stability. It is also more difficult to detect on two-dimensional radiographs due to the superimposition of intact root surfaces, underscoring the diagnostic value of CBCT [18]. Ericson and Kurol [18] were among the first to demonstrate that CBCT could identify resorption invisible on conventional radiographs, a finding subsequently confirmed by Alqerban et al. [2], who reported CBCT-detected resorption in 66.7% of cases compared with only 9.1% on panoramic radiographs.

The 45° angulation threshold has direct clinical implications for risk stratification and treatment planning. Canines angled ≥ 45° warrant heightened vigilance, more frequent monitoring, and consideration of early interceptive treatment. CBCT evaluation is recommended for palatally displaced canines when conventional radiographs suggest high angulation, to enable early detection and appropriate management, including extraction of primary canines, surgical exposure, or orthodontic traction [13,14,15].

Limitations include the retrospective design, single-examiner measurements, single-center recruitment, and absence of multivariable modeling to control for confounders such as canine-to-root proximity and root morphology. Additionally, the relatively small number of resorption cases (n = 17, 15.7%) constrains the statistical power for subgroup analyses and should be considered when interpreting the modest Spearman correlation coefficient (ρ = +0.243). The small resorption sample increases the risk of type II error in secondary analyses and limits the precision of the 45° threshold estimate. Furthermore, the vertical facial growth pattern was not assessed as a covariate in this study, despite evidence that it may modulate impaction predisposition [12]. Prospective multi-center studies incorporating multivariable analysis are warranted to validate the 45° threshold and develop comprehensive risk prediction models.

## 5. Conclusions

This CBCT-based study demonstrates a significant positive correlation between impacted maxillary canine angulation and adjacent lateral incisor root resorption severity (ρ = +0.243, *p* = 0.019). Canines angled ≥ 45° from the vertical reference line exhibit a 3.5-fold higher resorption rate (OR = 4.66, *p* = 0.012). The palatal crown position, angulation ≥ 45°, and unilateral impaction are significant risk indicators for lateral incisor root resorption. These findings support the routine use of CBCT-based angulation measurement for early risk stratification and treatment planning in patients with impacted maxillary canines.

## Figures and Tables

**Table 1:** Demographic and clinical characteristics of included patients.

Characteristic	Value
Total CBCT scans reviewed	296
Excluded (did not meet inclusion criteria)	34
Total patients included	262
Patients contributing evaluable canines	88
Total evaluable impacted canines	108
Sex ^†^, n (%)	
Female	51 (58%)
Male	37 (42%)
Age (years)—88 patients	
Mean ± SD	19.4 ± 9.6
Range	9–57
Laterality of impaction—88 patients, n (%)	
Unilateral right	35 (39.8%)
Unilateral left	32 (36.4%)
Bilateral	21 (23.9%)
Canine side—108 canines, n (%)	
Right (#13)	63 (58.3%)
Left (#23)	45 (41.7%)
Canine status—108 canines, n (%)	
Fully impacted	58 (53.7%)
Partially impacted	46 (42.6%)
Erupted but malpositioned	4 (3.7%)
Crown position—108 canines, n (%)	
Palatal	51 (47.2%)
Center	39 (36.1%)
Buccal	18 (16.7%)

^†^ Sex percentages calculated per canine (n = 108); bilateral cases (21 patients, 41 canines) contribute two canines each. IQR, interquartile range; SD, standard deviation.

**Table 2:** Distribution of lateral incisor root resorption grades (Modified Kaley & Phillips classification).

Resorption Grade	Description	n	%
Grade 0	No resorption—smooth root contour	91	84.3
Grade 1	Slight apical resorption (minor blunting of apex)	3	2.8
Grade 2	Moderate apical resorption (up to ¼ root length lost)	5	4.6
Grade 3	Severe apical resorption (>¼ root length lost)	3	2.8
Grade 4 *	Side resorption—lateral/cervical surface (direct crown–root contact)	6	5.6
Any resorption (Grade ≥ 1)		17	15.7
Apical resorption (Grades 1–3)		11	10.2
Side resorption (Grade 4)		6	5.6
Severe + side resorption (Grades 3–4)		9	8.3
Total		108	100.0

* Grade 4 is a morphologically distinct pattern (lateral surface resorption) separate from the apical resorption continuum of Grades 1–3. Pink cells = significantly higher angulation. Total n = 108 impacted canines.

**Table 3:** Canine angulation measurements stratified by resorption status, grade, and crown position.

Category	n	Mean ± SD (°)	Median (°)	IQR (°)
Overall (valid angle measurements)	93	34.8 ± 19.1	33.4	19.1–48.8
By Resorption Status				
No resorption (Grade 0)	78	32.7 ± 18.0	30.0	18.4–44.5
Any resorption (Grade ≥1)	15	45.5 ± 21.4	51.6	—
By Resorption Grade				
Grade 0 (no resorption)	78	32.7 ± 18.0	30.0	18.4–44.5
Grade 1 (slight apical)	3	53.0 ± 18.0	56.9	45.1–62.8
Grade 2 (moderate apical)	4	37.0 ± 32.6	33.9	15.1–55.8
Grade 3 (severe apical)	3	52.5 ± 9.2	54.0	48.3–57.4
Grade 4 (side resorption)	5	43.6 ± 21.0	51.6	35.9–53.7
By Crown Position				
Palatal	46	39.1 ± 17.8	39.2	—
Buccal	12	38.8 ± 25.5	35.1	—
Center	35	27.7 ± 16.5	22.8	—
By Angulation Threshold				
Angle < 45°	65	—	—	—
Angle ≥ 45°	28	—	—	—
Mann–Whitney U (resorption vs. no resorption): U = 809.5, *p* = 0.019 *|Spearman ρ (angle vs. grade): ρ = +0.243, *p* = 0.019 *				
Kruskal–Wallis (angle across crown positions): H = 8.244, *p* = 0.016 *|Post hoc: palatal vs. center *p* = 0.003 **; palatal vs. buccal *p* = 0.737 (ns); buccal vs. center *p* = 0.283 (ns).				

* *p* < 0.05; ** *p* < 0.01; ns, not significant. Pink cells = significantly higher angulation. IQR, interquartile range; SD, standard deviation.

**Table 4:** Root resorption prevalence by crown position, angulation threshold, and impaction pattern.

Group	Total n	Resorption Present, n (%)	No Resorption, n (%)
Crown Position			
Palatal	51	13 (25.5%)	38 (74.5%)
Buccal	18	2 (11.1%)	16 (88.9%)
Center	39	2 (5.1%)	37 (94.9%)
Total	108	17 (15.7%)	91 (84.3%)
Angulation Threshold			
Angle < 45°	65	6 (9.2%)	59 (90.8%)
Angle ≥ 45°	28	9 (32.1%)	19 (67.9%)
Impaction Pattern			
Unilateral	67	15 (22.4%)	52 (77.6%)
Bilateral	41	2 (4.9%)	39 (95.1%)
Total	108	17 (15.7%)	91 (84.3%)
Chi-square (crown position): χ^2^ = 7.258, df = 2, *p* = 0.027 * | Fisher’s exact (≥45° vs. <45°): OR = 4.66, *p* = 0.012 *			
Chi-square (unilateral vs. bilateral): χ^2^ = 4.634, df = 1, *p* = 0.031 * | Relative risk for ≥45°: 3.5-fold increase.			

* *p* < 0.05.

## Data Availability

The data supporting the findings of this study are available from the corresponding author upon reasonable request.
